# Oral Administration of* Lactobacillus plantarum* 299v Reduces Cortisol Levels in Human Saliva during Examination Induced Stress: A Randomized, Double-Blind Controlled Trial

**DOI:** 10.1155/2016/8469018

**Published:** 2016-12-22

**Authors:** Hannah Andersson, Cecilia Tullberg, Siv Ahrné, Kristina Hamberg, Irini Lazou Ahrén, Göran Molin, Mikael Sonesson, Åsa Håkansson

**Affiliations:** ^1^Food Hygiene, Department of Food Technology, Engineering and Nutrition, Lund University, P.O. Box 124, 22100 Lund, Sweden; ^2^Faculty of Odontology, Malmö University, 205 06 Malmö, Sweden; ^3^Probi AB, Ideon Gamma 1, 223 70 Lund, Sweden

## Abstract

*Objective*. To clarify the effect of* Lactobacillus plantarum* 299v on the salivary cortisol and salivary IgA levels in young adults under examination stress.* Design*. Forty-one students with an upcoming academic exam were included in a randomized double-blind, placebo-controlled study. The probiotic bacteria or the placebo product was administered in capsules once a day during 14 days. Saliva was collected and a perceived stress test was filled out at each sampling occasion. Saliva was collected for cortisol analysis by Electrochemiluminescence Immunoassay (ECLI) and salivary IgA was analysed by Enzyme-Linked Immunosorbent Assay (ELISA). Abundance of lactobacilli was evaluated by cultivation of saliva on selective medium and identification of* L. plantarum* 299v was done on randomly selected colonies by a random amplification of polymorphic DNA (RAPD) typing.* Results*. A significant difference in cortisol levels was found between the treatment group and the placebo group (*P* < 0.05), together with a significant increase in levels of lactobacilli in the treatment group compared with the placebo group (*P* < 0.001). No significant changes were found for salivary IgA.* Conclusion*. A probiotic bacterium with ability to reduce symptoms of irritable bowel syndrome (IBS) prohibited increased levels of the stress marker cortisol during the examination period. The registration number of the study is NCT02974894, and the study is registered at ClinicalTrials.gov.

## 1. Introduction

There is a connection between the digestive tract and physiological stress [1, 2] and salivary cortisol and salivary immunoglobulin A (salivary IgA) can be taken as markers of stress [3, 4]. Cortisol is a steroid hormone released by the adrenals in response to stress and increased levels have been related to suppression of the immune system [5]. Salivary IgA is part of the oral immune defence and the most abundant antibody in saliva, predominantly in the form of secretory IgA [6]. Minor salivary glands, positioned throughout the oral mucosa, secrete about 30–35% of all salivary IgA that occurs in adult whole saliva [7].

Probiotics are defined as “live microorganisms which when administrated in adequate amounts confer a health benefit to the host” [8].* L. plantarum* 299v has in previous studies shown a positive effect on symptoms of irritable bowel syndrome (IBS) such as bloating and pain [9, 10]. This strain has also been shown to counteract gut leakiness [11] which in turn could attenuate the Hypothalamic-Pituitary-Adrenal (HPA) axis response to stress [1]. No previous studies have been conducted on the effect of* L. plantarum* 299v on stress related conditions. However, it has been shown in rat pups stressed by separation from their mothers that a probiotic mixture of* Lactobacillus rhamnosus *R0011 and* Lactobacillus helveticus* R0052 significantly decreased the levels of corticosterone, the cortisol analogy in the blood of animals [12]. Also, rats subjected to an acute time-limited stressor became less stressed after treatment with* Lactobacillus farciminis* [1], along with decreased levels of lipopolysaccharides (LPS) in the blood [1, 12].

Not much is known about the effects of probiotic bacteria on stress markers in the saliva, in spite of the fact that saliva sampling is an easy and noninvasive method that does not cause the test person any harm or discomfort. This is particularly important when evaluating stress markers where an acute rise might be due to the sampling method and not to the acute stressor evaluated in the study. When it comes to probiotic effects on salivary IgA levels, most studies are related to the incidence of various respiratory infections [13, 14], and, in a previous study, it was reported that the secretory IgA levels in stools increased in a group of children receiving a product containing* L. rhamnosus* HN001 compared to a placebo group [13]. Similar results were obtained with the strain* L. rhamnosus* GG [14].

Chronic stress is related to increased cortisol levels, but whether cortisol is a causative agent for the physiological stress reactions or just a consequence of it is open for discussion [15]. The release of cortisol is a delayed peripheral response to an acute stress [3] and it is suggested that cortisol exerts both stimulatory and suppressive actions on the body's stress response. An example of a stimulatory action is the triggering of the cardiovascular activation [16]; however, in response to, for example, a haemorrhage stressor, cortisol is suppressing the diminished fluid volume response by inhibiting the initial rapid secretion of vasoconstrictive stress hormones [16]. Regarding the immune regulating properties of cortisol, its secretion is suggested to protect against the “overshoot” of the immune response otherwise happening in response to stress [16]. However, it is not yet clear what purposes the increased level of cortisol serves during the body's response to stress [16].

In the present study the main goal was to clarify the effect of the probiotic bacteria* Lactobacillus plantarum* 299v on the salivary cortisol level in young adults under examination stress. Salivary IgA from minor glands and in whole human saliva was measured as alternative markers of stress. The hypothesis was that the levels of cortisol would be reduced or stabilized in the test group, in comparison to the placebo group where the cortisol levels were expected to increase due to the examination induced stress. By contrast, the salivary IgA levels were expected to increase in the test group in comparison to the placebo group.

## 2. Materials and Methods

### 2.1. Study Design

In the present randomized, double-blind placebo-controlled trial, volunteering students at Lund University, in the ages between 18 and 30 years, participated during their exam period. Exclusion criteria were pregnancy as well as intake of antibiotics and temporary cortisone medication during the study period. A power calculation was conducted in order to decide the amount of subjects and 40 individuals were regarded as sufficient. 42 healthy young adults volunteered to the study, 28 females and 14 males, and all participants gave their signed informed consent for the study. After one male drop-out, 41 individuals completed the whole experimental period. 21 were given the test product in a daily dose of 1 × 10^10^ colony forming units of* L. plantarum* 299v in capsules (*L. plantarum *299v, potato starch, hydroxypropyl methyl cellulose, and magnesium stearate, (Probi AB, Lund, Sweden)) and 20 were given placebo capsules, containing the same ingredients as the test product with the exception of the bacteria. Before starting the experimental period, there was a two-week wash-out period when the participants were instructed to avoid intake of probiotic products. Intake of the capsule occurred thereafter once a day after lunch and the subjects were asked not to eat, drink, smoke, or take snuff for one hour after the intake. The participants were also instructed to either chew on the capsule until the powder was dissolved in the mouth (minimum 30 sec) or empty the capsule on a spoon and chew the powder for 30 sec before ingesting. The reference samples for all participants were collected on day 0, before any intake of the product. The study continued for 14 days and the starting date was individually decided for all the participants, within a span of 1.5 weeks.

### 2.2. Psychological Assessment

A psychological assessment was included in order for the test subjects to evaluate their self-perceived stress. A questionnaire with 30 questions developed by Levenstein et al. [17] was used and the obtained lower score indicates a lower degree of stress and vice versa. An index giving a number between 0 and 1 was calculated using the following [17]:

(1) $$\begin{array}{c} Index=\frac{\sum \left(Question\;\;1\text{–}30\right)-30}{90}. \end{array}$$

### 2.3. Saliva Sampling

Minor gland saliva was collected from the lower labial mucosal areas as described earlier [18, 19] and whole saliva was harvested at the same sampling day. To reduce the influence of circadian variations in saliva secretion during collection, all sampling was made between 3 and 5 pm. All samples were collected on day 0 and day 14, and, on days 5 and 10, only cortisol samples were taken. The participants were instructed not to eat, drink, take snuff, smoke, brush their teeth, or do any kind of exercise one hour prior to sampling time. Alcohol was also prohibited twelve hours before the sample collection appointment.

### 2.4. Cortisol Analysis

Sampling of saliva for the cortisol analysis was performed with a Salivette® for cortisol (Sarstedt, Germany) and it was done after collection of saliva for IgA analysis. The participants were instructed to circulate the swamp in the mouth for 2–5 min, until it was filled with saliva and then carefully spit it back into the tube. The tubes were put on ice and later frozen at −20°C until analysis. The cortisol analysis was conducted by Labmedicin Skåne (the university and regional laboratories in Region Skåne, Sweden) and was performed according to accreditation. The method used was a one-step competition assay with the detection technique Electrochemiluminescence Immunoassay (ECLI) based on ruthenium (Ru) derivate [20].

### 2.5. Salivary IgA Analysis

Minor gland saliva was collected from the lower lip by a SialoPaper® strip (Oraflow Inc., New York, USA) and gently placed on mucosa for 60 s and the salivary volume within the strip was measured by a Periotron® 8000 (Oraflow Inc., New York, USA). The SialoPaper strip was put in 1 mL of PBS (1.5 mL PP, Sarstedt, Germany; Phosphate Buffered Saline, Dulbecco A, Oxoid, England) on ice and stored at −80°C until analysis. The unstimulated whole saliva was collected after sampling of minor gland saliva. The participants sat, leaning slightly forward, and let the saliva drain into a 2 mL micro tube with the help of a sterile straw (1 mL sterile disposable pipettes, Kemikalia, Sweden; 2 mL PP, Sarstedt, Germany). All samples were stored at −80°C until analysis.

The concentration of IgA secreted in the saliva is relative to the total protein amount and therefore the unit % IgA/total protein is chosen to represent the result of the whole saliva analysis. This is relevant since the secretion of IgA changes with the salivary flow rate, which in turn changes with stimuli on the autonomic nervous system, for example, acute stress, chronic stress, and resting. This change in flow rate is accounted for by measuring the % IgA/total protein in saliva, which makes it an accurate method when comparing data from different test subjects [21].

In order to analyse the salivary IgA concentration, a modified sandwich-ELISA was performed according to Krzywkowski et al. [22], using *α*1- and *α*2-chain-specific goat anti-human IgA (5 *μ*g/mL, Sigma I0884; Sigma Chemical Co., St Louis, MO, USA) conjugated to alkaline phosphatase (Sigma A9669; diluted 1 : 30,000 in PBST). The total protein concentration was analysed by a quick start protein assay (BIO-RAD) [19, 22].

The salivary IgA in the minor gland saliva was expressed as mg/100 mL. The analysis of salivary IgA was conducted at the Faculty of Odontology at Malmö University (Malmö, Sweden).

### 2.6. Plate Count of Lactobacilli

In order to analyse the oral lactobacilli the test subjects chewed on a paraffin pellet (Orion Diagnostics) to stimulate the secretion of saliva, and collection was done in the same way as for salivary IgA in whole saliva. The collection of this saliva sample was done at the end of each sampling occasion, since this sampling method gives strongly stimulated saliva, contrary to the other methods. The tubes were put on ice and 0.25 mL of the saliva sample was transferred to another sterile micro tube (1.5 mL PP, Sarstedt, Germany) which contained 0.5 mL freezing media. Both tubes were saved in −80°C until analysis.

Abundance of lactobacilli was evaluated by plate count. The samples were diluted and spread on Rogosa agar plates (Oxoid, England) and the plates were incubated at 37°C for 72 hours under anaerobic conditions (2.5 L, AnaeroGen™, Oxoid). Two colonies from each plate were randomly picked and recultivated. The isolates were frozen and stored in freezing media at −80°C.

### 2.7. Identification of* L. plantarum *299v

For DNA extraction of each isolate the samples were thawed and vortexed shortly before addition of 1 mL of sterile MilliQ water and centrifugation at maximum speed (14 800 ×g, 1 min). The supernatant was removed and another 0.25 mL of sterile MilliQ water was added. The remaining cell extract was smoothly grinded with sterile Assistent® glass beads (Ø 2 mm, Glaswarenfabrik Karl Hecht, Germany) in a shaker (6°C, 30 min) and all samples were then used in a random amplification of polymorphic DNA (RAPD) reaction, to study the occurrence of* L. plantarum* 299v. Mastermix for 35 samples including 175 *μ*L buffer (Qiagen, Germany), 35 *μ*L dNTP (Roche Diagnostics, Germany), 35 *μ*L Primer 73 (Qiagen, Germany), and 8.75 *μ*L Taq-Polymerase (Qiagen, Germany) was prepared. 7.25 *μ*L of the mastermix was added to each PCR tube (Gene Amp 0.5 mL, Applied Biosystems, Singapore) together with 42.75 *μ*L of H_2_O and 1 *μ*L of the supernatant of the samples.

The RAPD reaction was performed in a PCR machine (Perkin Elmer, DNA Thermal Cycler, USA) according to Quednau et al. [23]. Gels for the gel electrophoresis were prepared by adding 0.75 g Agarose DNA Grade Electran® (VWR, Belgium) into 50 mL TB buffer; then the solution was heated until boiling and poured into gel tanks to cool. The gels were finally put into gel trays with TB buffer. 2–5 *μ*L of each sample was added into each well together with 3 *μ*L of loading buffer before running the electrophoresis (100 V, 1 h). Gel Redx3 (Biotium Inc., USA), 1 mL in 99 mL dH_2_O, was used for 20 min to colour the gels and photos were taken according to Quednau et al. [23].

### 2.8. Statistical Calculations

The data was evaluated statistically using a Kruskal-Wallis One-Way Analysis of Variance (ANOVA) on Ranks or a Mann–Whitney Ranks Sum test when appropriate. A *t*-test was used for pairwise comparison of the absolute IgA concentrations as well as of the absolute cortisol levels. The statistical tools were Sigma Plot (11.0 and 12.0) and Excel 2013. Moreover, an incidence test regarding the occurrence of* L. plantarum* 299v was performed with Fisher Exact test, using QuickStat (2.6). Data was presented as median and interquartile range, as well as mean and standard deviation. The minimum and maximum values for the cortisol samples were deleted to remove the impact of outliers, since a clear deviant could be seen in the data. This only concerned the cortisol samples. Values belonging to participants that did not fulfil all the study requirements were controlled for and were not found to deviate. Their results were therefore included.

### 2.9. Ethical Permission

The study was approved by the Regional Ethical Review Board in Lund (Ref 2013/166) and the ethical considerations included the benefits and risks with the used product and the possibility of already existing personal relations between the responsible test personnel and the test subjects conducting the study.

## 3. Results

### 3.1. Evaluation of the Psychological Assessment

The psychological assessment showed no significant difference of perceived stress between the groups, and there was additionally no significant difference within each group when comparing the different sampling days.

### 3.2. Cortisol Levels in Saliva

**Figure 1:**
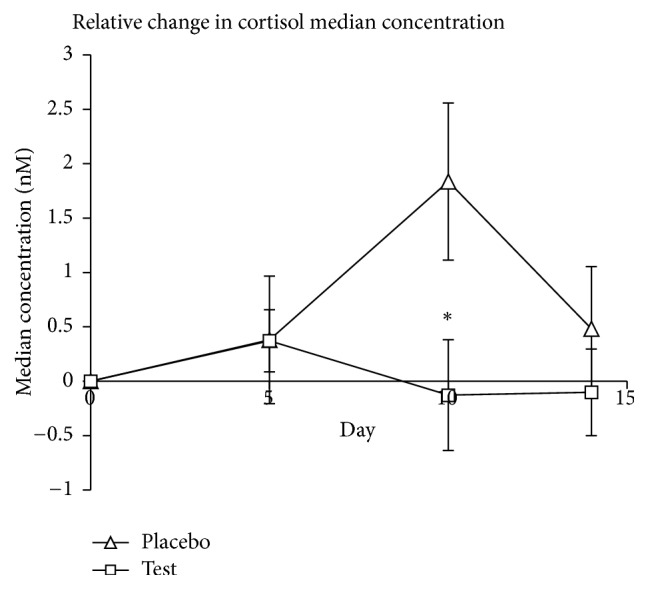
The difference in median cortisol values (nM) compared to reference values (day 0). Asterisk (*∗*) denotes a significant difference (*P* < 0.05) compared to the placebo group on day 10.

The absolute cortisol levels (nM) on the reference day (day 0) were 9.18 (±2.50) for the placebo group and 9.47 (±2.96) for the test group, with no significant difference between the two groups (*P* = 0.73). When the relative change in median cortisol levels was compared to a reference value on day 0 (baseline), a significant difference was found between the groups on day 10 (*P* < 0.05; Figure 1). No significance was found within the groups.

### 3.3. IgA Concentrations in Saliva

**Figure 2:**
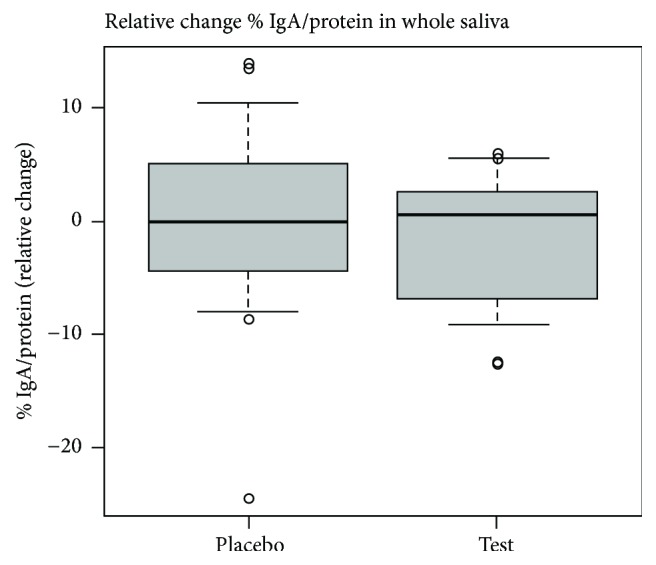
The relative change in whole saliva median % IgA/total protein conc. values (day 14-day 0) and the 25th and 75th percentile (box) as well as the 10th and 90th percentile (error bars). The dots show outliers. All data is included.

**Figure 3:**
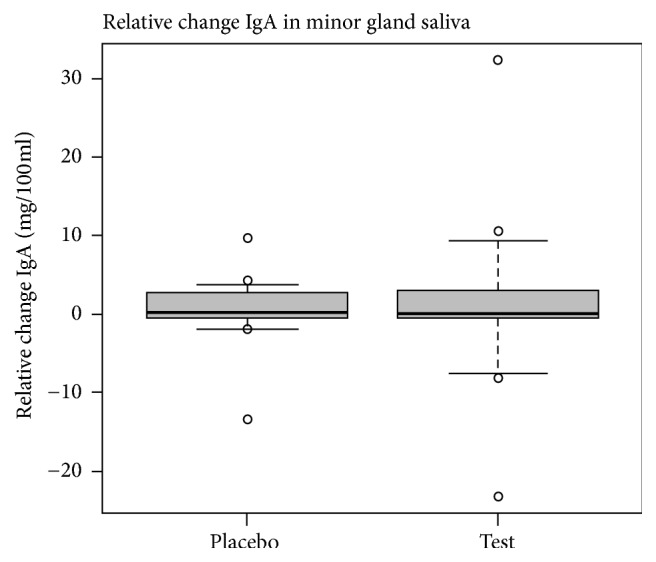
Relative change in median minor gland IgA sample, shown as mg/100 mL, comparing day 0 to day 14. The box shows the 25th and 75th percentiles and the error bars the 10th and 90th percentiles. The dots show outliers. All data is included.

The absolute IgA levels (mg/100 mL) in the minor gland saliva on the reference day (day 0) were 1.99 (±3.76) for the placebo group and 4.80 (±9.17) for the test group, with no significant difference between the two groups (*P* = 0.23). The absolute IgA levels (% IgA/total protein) in the whole saliva at the reference day (day 0) were 25.3 (±7.37) for the placebo group and 27.1 (±12.2) for the test group, with no significant difference between the two groups (*P* = 0.58). In contrast to the changing cortisol levels, the median salivary IgA concentrations in whole saliva or saliva from the minor glands were not affected by the probiotic consumption; that is, no significant difference was shown between the probiotic group and the placebo group (Figures 2 and 3, resp.).

### 3.4. Viable Count of Lactobacilli and Identification of* L. plantarum* 299v

**Figure 4:**
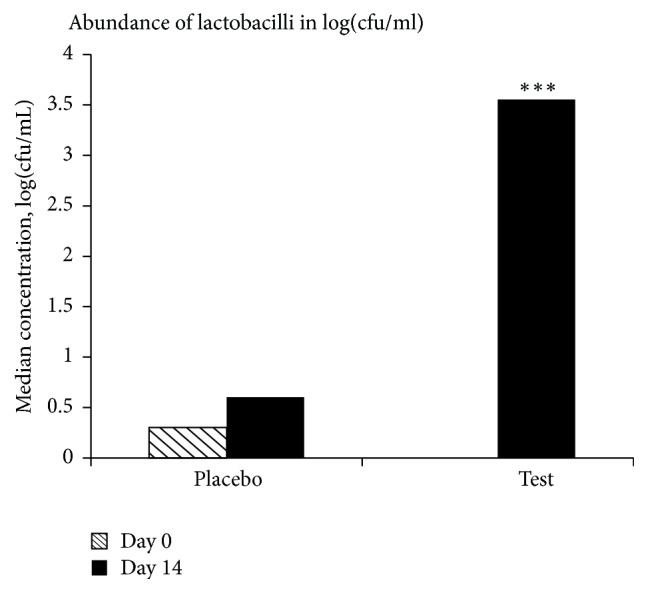
Abundance of lactobacilli in log(cfu/mL) showing the median values. The asterisks (*∗∗∗*) denote a significant difference (*P* < 0.001) compared to the placebo group on day 14 and the test group on day 0.

On day 14 there was a significant increase in the abundance of lactobacilli in saliva of the test subjects consuming probiotics in comparison to those given the placebo product (*P* < 0.001). The increase was also significant (*P* < 0.001) within the probiotic group when comparing the baseline with the final sampling day (*d* = 14, Figure 4). The incidence of isolates identified as* L. plantarum *299v was significantly increased on day 14 in persons with detectable levels of lactobacilli, both when compared to the placebo group and to the baseline (*P* < 0.01).

## 4. Discussion

Most participants had their exam before the end of the study, resulting in an apparent, but not significant (*P* = 0.08), peak of perceived stress on day 10 for the placebo group. This coincides with the peak in salivary cortisol concentration in the placebo group on day 10 (Figure 1). In contrast, no peak was seen in the probiotic group, suggesting that the probiotic consumption had an effect on stress seen as the secretion of cortisol from the adrenals. On day 14, many of the volunteers had finished their exams and a decrease in cortisol could therefore be expected, since cortisol responds relatively quickly to acute stressors.

Academic stress, here in the form of an exam period, cannot be considered as chronic stress, since the participants can see an end of the stressful period. It is therefore suggested that academic stress is more similar to acute stress with regard to the stress response. The cortisol levels of the present study were in the same range as what has previously been reported for acute stress [3].

Chronic stress is related to a sustained increased secretion of cortisol. It is recently debated whether it is the actual increased levels of cortisol or rather how the target tissues respond to the increased levels that is the cause of many diseases and symptoms associated with chronic stress [15]. What is known is that a prolonged increase in blood glucose levels may lead to muscle wastage, fat accumulation, and redistribution and possible diabetes. Hence, a possible target for probiotics could be chronically stressed individuals, where the sustained elevated levels of cortisol increase their risk for developing diseases connected to chronic stress [3, 16].

There are generally large individual differences in the measured levels of salivary IgA, which muddles attempts to see differences between small groups of individuals. However, the median values in the different groups correspond well to those reported by others [19, 21]. The present results from the whole saliva samples showed that the values between placebo and test group did not differ significantly from each other and that the median values in both groups were similar. Looking at the relative change in each group, the median value was slightly positive for the probiotic group, whereas it was slightly negative in placebo group. Comparing with the 25th and 75th percentiles, an increase in a major part of the detected salivary IgA levels is observed in the placebo group, which makes it hard to draw any conclusions (Figure 2). Stressors causing chronic stress have been connected to decreased levels of salivary IgA [24, 25], while acute stress has been shown to increase the levels [25], which indicates an activation of the immune response. It could therefore be suggested that the stressor in this study is more likely to be acute than chronic.

Previous studies have shown a trend suggesting that probiotics can increase the salivary IgA levels during stress [13, 14, 26]. However, the test persons of the present study only had an intake of probiotics during a two-week period, and even though there was a significant higher level of lactobacilli in this group, it could possibly be that the study period was too short to see clear changes on an individual level. Due to the large individual differences in salivary IgA levels, an increased number of participants in each group would also have been desirable.

The salivary IgA levels in the minor gland saliva is found to be higher than reported by others, which might be due to individual differences or due to the study design of this work. For example, a certain stress level and stress type was applied to the participants, which was not applied in the study done by Sonesson et al. [19]. The data from the minor gland saliva showed that median values were higher in the test group than in the placebo group, but the difference between the groups was not significant. The increase appears to be larger in the group given probiotics compared to the placebo group, but the relative change in median value is approximately the same in the two groups. However, the error bars indicate large variations within the group given probiotics, which might explain the contradiction (Figure 3).

Brief naturalistic stress, including academic stress, is expected to be somewhere in between acute and chronic stress regarding increase or decrease of stress markers. Since the stressor is hard to define in regard of the type of stress that is applied and since the individual stress applied to each participant might have been different, the insignificant differences found are in line with the theory for how IgA can reflect stress. It might have been easier to observe changes in salivary IgA if the study period would have been longer, or if additional samples would have been collected one or two weeks after the study, since the IgA levels have been seen to decrease also some time after the experienced stress in other studies [3, 27]. Another explanation could be that even though the participants were told to write down any changes in their health state (e.g., cold incidents), this was neither controlled for, nor compared with outliers in the salivary IgA data. Colds and other respiratory infections would probably cause an increase in salivary IgA, as IgA is part of the immune system's first line of defence against pathogens.

There was a significant increase in the levels of lactobacilli for the probiotic group, also in comparison to the placebo group, showing that the administrated bacteria could survive in the oral cavity (Figure 4). This was also supported by the outcome of the RAPD-typing, indicating the occurrence of* L. plantarum *299v in the samples where the levels of lactobacilli were above the detection limit. Thus, there seems to be a link between increased presence of the probiotic bacteria and the decreased cortisol levels in saliva.

## 5. Conclusions

There is a significant difference in cortisol secretion response between test persons given probiotics or placebo, possibly in relation to stress. A significant increase was moreover seen in the levels of lactobacilli for the probiotic group in comparison to the placebo group. In contrast, no significance was found for salivary IgA.
